# Interest in Digital Peer-Delivered Interventions and Preferences to Improve Pain Self-efficacy and Reduce Loneliness Among Patients With Chronic Pain: Mixed Methods Co-design Study

**DOI:** 10.2196/41211

**Published:** 2023-04-14

**Authors:** Eloise Yates, Lisa Buckley, Michele Sterling, Tegan Cruwys, Claire E Ashton-James, Renee Rankin, Rachel A Elphinston

**Affiliations:** 1 RECOVER Injury Research Centre The University of Queensland Brisbane Australia; 2 Australian Pain Management Association Brisbane Australia; 3 School of Public Health The University of Queensland Brisbane Australia; 4 National Health and Medical Research Council Centre for Research Excellence in Road Traffic Injury Recovery The University of Queensland Brisbane Australia; 5 Research School of Psychology The Australian National University Canberra Australia; 6 Sydney Medical School The University of Sydney Sydney Australia; 7 Metro South Addiction and Mental Health Service Metro South Health Hospital and Health Service Brisbane Australia

**Keywords:** chronic noncancer pain, pain, self-efficacy, peer support, peer-led intervention, peer-led, peer delivered, peer intervention, pain self-efficacy, social connectedness, social support, loneliness, lonely, social isolation, co-design, participatory design, qualitative research, digital health

## Abstract

**Background:**

Two important factors that prolong and exacerbate chronic noncancer pain (CNCP) and disability are low pain self-efficacy and loneliness. Yet, few interventions have shown long-term sustained improvements in pain self-efficacy, and there are no evidence-based treatments that target social connectedness in people living with CNCP. More effective and accessible interventions designed to target self-efficacy and social connectedness could ease the burden of CNCP.

**Objective:**

To co-design accessible interventions to increase pain self-efficacy, social connection, pain-related outcomes, and quality of life, this study explored patients’ interest and preferences for digital peer-delivered interventions for CNCP as well as implementation barriers and enablers.

**Methods:**

This cross-sectional mixed methods study was part of a larger longitudinal cohort study. Adult Australian residents (N=186) with CNCP diagnosed by a medical professional or pain specialist were included. Participants were initially recruited through advertising on professional pain social media accounts and websites. Questions examined whether patients were interested in digital peer-delivered interventions and their preferences for specific features (eg, Newsfeed). Pain self-efficacy and loneliness were assessed using validated questionnaires, and the association between these factors and interest in digital peer-delivered support was explored. Open-ended questions explored implementation barriers, enablers, and suggestions for consideration in intervention design.

**Results:**

There was interest in accessing digital peer-delivered interventions, with almost half of the sample indicating that they would access it if it was available. Those who indicated an interest in digital peer interventions reported both lower pain self-efficacy and greater loneliness than those who were not interested. Intervention content that incorporated education, links to health services and resources, and delivery of support by peer coaches were the most frequently preferred intervention features. Three potential benefits were identified: *shared experience*, *social connection*, and *shared pain management solutions*. Five potential barriers were identified: *negative focus on pain*, *judgment*, *lack of engagement*, *negative impact on mental health*, *privacy and security concerns*, and *unmet personal preferences*. Finally, there were 8 suggestions from participants: *moderation of the group*, *interest subgroups*, *professional-led activities*, *psychological strategies*, *links to professional pain resources*, *newsletter*, *motivational content*, *live streaming*, and *online meetups*.

**Conclusions:**

Digital peer-delivered interventions were of particular interest to those with CNCP who had lower levels of pain self-efficacy and higher levels of loneliness. Future co-design work could tailor digital peer-delivered interventions to these unmet needs. Intervention preferences and implementation barriers and enablers identified in this study could guide further co-design and the development of such interventions.

## Introduction

Neck and low back pain are the leading causes of disability burden in Australia and worldwide [1]. The economic costs of chronic noncancer pain (CNCP) are significant [2] as are the personal costs of concomitant difficulties with day-to-day functioning, loneliness, mental health problems, and overall poor quality of life [3]. Two important factors associated with persistent CNCP and disability are low pain self-efficacy and loneliness [4-7]. More effective and accessible treatments targeted to these modifiable risk and protective factors could better meet the needs of patients and help reduce the increasing burden of CNCP.

A lack of confidence in carrying out daily activities despite the pain (low pain self-efficacy [8]) more strongly predicts pain disability than other important pain-related factors such as fear-avoidance beliefs [4,9]. Lower pain self-efficacy is also associated with increased use of both pain clinics and emergency departments [10]. Yet, few of the current approaches to improving pain self-efficacy have shown long-term sustained improvements. Meta-analytic evidence from a systematic review of 60 randomized controlled trials (12,415 patients with chronic musculoskeletal pain) found that multicomponent, cognitive behavioral, and exercise interventions had small effects on pain self-efficacy at 3 months, and these improvements were not sustained beyond 3 months; self-management interventions were not found to improve pain self-efficacy at any time point [11].

The benefits of improved physical and psychological health and longevity that arise from feelings of social connectedness are widely recognized [12,13]. Individuals with stronger social connections have a 50% increased likelihood of survival than those with weaker social connections—so much so that the influence of social connectedness on the risk of death is comparable with well-established risk factors for mortality such as smoking and alcohol use, and exceeds that of physical inactivity and obesity [13]. The related concept of social support, derived from social connections in times of need [12] includes emotional support (eg, care and empathy), appraisal support (eg, being listened to), instrumental support (practical help and getting to and from appointments), and informational support (eg, receiving advice) [14]. For individuals with CNCP, access to social support improves emotional and psychological well-being [15], recovery from co-occurring depression [16], pain management, coping skills, and an individual’s ability to self-manage their condition [17]. Conversely, the consequences of inadequate social connections and increased loneliness include poorer physical health and lower overall well-being, particularly among those with CNCP [5,18]. But there are very few treatments for CNCP that address the need for social support, and those interventions that provide social support are inaccessible to the majority of people with chronic pain [19].

Peer-to-peer support presents an opportunity for intervention to improve pain self-efficacy within an environment of collective support [20,21]. Digital group peer support enables users to share common experiences and successes, engage in collective problem-solving, and provide mutual support, which are all important ways to increase self-efficacy [22,23]. In CNCP samples, peer-delivered interventions have shown some positive effects on pain, quality of life, and emotional well-being compared with usual care [24]. A patient’s sense of social connection with the treatment group is also a mechanism of change in chronic pain groups [22]. Beneficial effects of digital or web-based peer-delivered interventions on self-efficacy have also been found across a range of chronic conditions [23]. Co-design of digital peer support interventions could further increase the effectiveness of digital peer-delivered interventions and accelerate translation to clinical practice [25].

Qualitative studies (N=20) [23] illustrate patients’ positive experiences of participating in web-delivered peer support interventions. These experiences include improved compassion and attitude toward living with their condition (eg, patients felt less alone in their difficulties), access to information about treatment options, and an increased sense of empowerment. However, a lack of available time or an inability to access the internet, a sense of not fitting in with the group, and a need for more condition-specific information were described as barriers. Enablers included intervention flexibility, appropriate session length, the usability of the technology, and remote access. Further insights into the barriers and enablers of digital peer support may enhance intervention engagement and adoption particularly to promote pain self-efficacy.

This study aimed to examine patient interest in digital peer-delivered interventions for CNCP and potential factors associated with interest (with a focus on pain self-efficacy and loneliness), and explore preferences for peer-delivered support as well as implementation barriers and enablers. It was hypothesized that there would be patient interest in digital peer support interventions for CNCP, but that patients with lower pain self-efficacy and self-reported feelings of loneliness would be particularly interested. Findings will inform the co-design of new and innovative digital peer support interventions to increase pain self-efficacy, pain-related outcomes, and quality of life among those with CNCP.

## Methods

### Study Design, Setting, and Participants

This cross-sectional study conducted in Australia is part of a larger longitudinal cohort study that aims to examine psychosocial factors associated with pain, mental health, and substance use disorders. At the end of the wave 1 survey (2019), participants were invited to provide contact information if they wished to participate in future research (84% accepted). Data presented in this study are from wave 3 collected between November 2021 and January 2022. Participants were initially recruited through advertising on professional pain social media accounts and websites (eg, Chronic Pain Australia). Individuals who expressed an interest were provided with a link to the web-based questionnaire (completed in Research Electronic Data Capture [REDCap], hosted by The University of Queensland) [26,27]. Potential participants completed eligibility questions, and if eligible, they proceeded to the web-based questionnaire. Individuals were eligible to participate if they self-reported current CNCP for 3 months or longer diagnosed by a medical professional or pain specialist, resided in Australia, and were 18 years or older at the time of the survey (wave 3).

### Ethics Approval

All participants provided informed consent. Participants were eligible to enter the draw for 1 of 2 Aus $100 (US $72) gift cards. The web-based survey took approximately 30 minutes to complete, and the data collected were deidentified for analysis. The study was approved by the relevant institutional review board (approval number: 2019000610, The University of Queensland Human Research Ethics Committee).

### Measures

Participants completed validated measures of their pain experience and related psychosocial factors and purpose-built questions examining digital peer support intervention needs and preferences. Measures relevant to this study’s aims are described below.

#### Demographics

Participants were asked for their date of birth, gender, current employment status (employed vs unemployed), highest level of education (university degree vs no university degree), and current relationship status (in a relationship vs not in a relationship). They were also asked to report on any current mental health diagnoses.

#### Pain Experience

Participants were asked about the physical location of pain and the original cause. They were asked to report on the number of days in the past 90 in which they experienced pain. Participants also completed the Brief Pain Inventory [28] to assess pain severity (4 items) and pain interference (7 items). Mean scores are computed from responses to 11-point Likert-type scales for pain severity (0=“no pain” to 10=“pain as bad as you can imagine”) and pain interference (0=“does not interfere” to 10=“completely interferes”). The Brief Pain Inventory has demonstrated evidence of reliability and validity in CNCP populations [29].

#### Pain Self-efficacy

The Pain Self-Efficacy Questionnaire [30] includes 10 items measured on a 7-point Likert-type scale (0=“not at all confident” to 6=“completely confident”). Scores are summed with higher scores reflecting greater pain self-efficacy. The Pain Self-Efficacy Questionnaire has shown internal consistency and test-retest reliability [30].

#### Loneliness

The Short-Form UCLA Loneliness Scale (UCLS-8) includes 8 items measured on a 4-point Likert-type scale (1=“Never” to 4=“Always”). Two items are reversed scored. Scores are summed with higher scores indicating a greater degree of loneliness. The UCLS-8 has demonstrated good reliability and validity [31].

#### Attitudes Toward Digital Peer Support Intervention

Participants were asked about their interest in participating in a digital peer support group for CNCP if it were available, and their intervention preferences (newsfeed, private chat, questionnaires, monitoring and support by trained peer coaches, and educational information or tips). Responses were recorded on a dichotomous scale (yes or no). Three open-ended response questions were included to examine the potential benefits, barriers, and recommendations for a digital CNCP peer support group. A full summary of the purpose-built intervention questions can be found in Multimedia Appendix 1.

### Data Analysis

All statistical analyses were performed using SPSS (version 27.0; IBM Corp). Initial descriptive statistics were calculated to describe the demographic and clinical characteristics of the sample. Frequency analyses and cross-tabulations were used to examine interest in the digital peer support intervention and preferences. Two one-way between groups ANOVAs were conducted to examine whether interest in digital peer support interventions was associated with pain self-efficacy and loneliness.

Qualitative analysis was performed using NVIVO (Version 12.5.0; QSR International). Comments from the 3 open-ended questions were thematically coded using data-driven codes. Coding undertook 2 iterations until all comments met a criterion for a code. Codes were then organized into higher-order themes. Themes were refined through discussion (EY and RE).

## Results

### Preliminary Statistics and Sample Characteristics

There were 225 respondents to the wave 3 questionnaire. Data were excluded from 39 participants due to missing data on all questions about a digital peer support intervention. A missing variable analysis showed data were missing completely at random (Little’s Missing Completely At Random test [MCAR] χ^2^_136_=18.06; *P*=.72). Missing data were handled using listwise deletion.

The final sample included 186 participants (130 female, 41 male, and 2 other) ranging in ages from 21 to 86 years. Table 1 shows the patient demographic characteristics, and Table 2 shows relevant clinical characteristics. Participants reported clinical levels of CNCP (defined as mean scores ≥4 [32]) and relatively low levels of pain self-efficacy (defined as mean scores <40 [8]; 76% of the sample had scores <40).

**Table 1:** Patient demographic characteristics (N=186).

Patient characteristics		Values^a^
Age (years), mean (SD)		54.9 (15.74)
**Gender, n (%)**		
	Female	130 (69.9)
	Male	41 (22.0)
	Other	2 (1.1)
**Education, n (%)**		
	High school	38 (20.4)
	Trade/diploma	52 (28.0)
	Bachelor’s degree	41 (22.0)
	Postgraduate degree	43 (23.1)
**Employment, n (%)**		
	Full-time	36 (19.4)
	Part-time/casual/contract	24 (12.9)
	Student	11 (5.9)
	Unemployed	22 (11.8)
	Retired	65 (34.9)
	Volunteer	7 (3.8)
	Other	21 (11.3)
**Relationship status, n (%)**		
	Single	32 (17.2)
	Relationship	8 (4.3)
	De facto	15 (8.1)
	Married	82 (44.1)
	Divorce	20 (10.8)
	Separated	5 (2.7)
	Widowed	10 (5.4)
**Location of chronic pain, n (%)**		
	Head/face	47 (25.3)
	Neck	86 (46.2)
	Shoulder/upper limbs	96 (51.6)
	Back/spine/sacrum	120 (64.5)
	Lower limbs	90 (48.4)
	Whole body	43 (23.1)
	Abdomen/pelvis/groin	63 (33.9)
**Cause of pain, n (%)**		
	Road traffic crash injury	6 (32.0)
	Injury at work	26 (14.0)
	After surgery	10 (5.4)
	Injury in another setting	21 (11.3)
	Medical condition other than cancer	53 (28.5)
	No obvious cause	27 (14.5)
	Other	29 (15.6)

^a^Total percentages do not add to 100 where multiple options could be selected (employment and pain location).

**Table 2:** Clinical characteristics of the sample.

Variable	Participants, n	Score, mean (SD)
Pain severity	174	5.39 (2.12)^a^
Pain interference	174	5.41 (2.33)^a^
Pain self-efficacy	174	31.17 (15.20)^b^
Loneliness	186	17.25 (4.19)^c^

^a^Score range 0-10.

^b^Score range 0-60.

^c^Score range 8-32.

### Interest in Digital Peer Support Interventions

Almost half (n=88, 47.3%) of patients reported that they would be interested in accessing digital peer support if it was available. Interest in digital peer support interventions was significantly associated with pain self-efficacy, with those indicating that they were interested in reporting lower levels of pain self-efficacy (*F*_1,172_=5.62; *P*=.02). Similarly, interest in digital peer support interventions was significantly associated with loneliness, such that those who were interested reported higher levels of loneliness (*F*_1,184_=13.74; *P*<.001; see Table 3 for means and frequencies).

**Table 3:** Means and SDs for pain self-efficacy and loneliness and interest in peer support.

Variable		Participants, n	Score, mean (SD)
**Pain self-efficacy^a^**			
	Yes	90	28.23 (15.61)
	No	84	33.78 (14.32)
**Loneliness^b^**			
	Yes	98	18.41 (4.08)
	No	88	16.20 (4.02)

^a^Score range 0-60.

^b^Score range 8-32.

#### Patient Preferences: Intervention Content and Delivery

Of the 88 participants who reported that they would be interested in a digital peer support intervention, the majority indicated preferences for educational content (n=71, 80.7%) and support from peer coaches (n=72, 81.8%). Most indicated an interest in receiving information and links to other health services (n=61, 69.3%) and more than half (n=49, 55.7%) were interested in content delivered via a newsfeed, access to questionnaires with feedback, and a private chat function.

Interestingly, almost one-quarter of the 98 participants who reported they were *not* interested in the intervention nevertheless indicated preferences for educational content (n=21, 21.4%), links to other health services (n=24, 24.5%), and support by coaches (n=21, 21.4%). Some indicated that they would like to complete questionnaires and receive feedback (n=13, 13.3%), content delivered via a newsfeed (n=11, 11.2%), and a private chat function (n=10, 10.2%). See Table 4 for a summary of interest in each content and delivery feature for those who were interested and those who were not and the proportion of the total sample.

**Table 4:** Summary of intervention preferences for the total sample and those interested or not interested in digital peer support.

			Interested in peer support (n=88), n (%)					Not interested in peer support (n=98), n (%)					Total sample (N=186), n (%)		
			Yes		No		Yes			No		Yes			No
**Content**															
	Education	71 (80.7)		17 (19.3)		21 (21.4)			77 (78.6)		101 (54.3)			85 (45.7)	
	Links to health services	61 (69.3)		27 (30.7)		24 (24.5)			74 (75.5)		85 (45.7)			101 (54.3)	
	Newsfeed	49 (55.7)		39 (44.3)		11 (11.2)			87 (88.8)		60 (32.3)			126 (67.7)	
**Delivery**															
	Support by coaches	72 (81.8)		16 (18.2)		21 (21.4)			77 (78.6)		93 (50.0)			93 (50.0)	
	Questionnaires/ feedback	51(58.0)		37 (42.0)		13 (13.3)			85 (86.7)		64 (34.4)			122 (65.6)	
	Private chat	51 (58.0)		37 (42.0)		10 (10.2)			88 (89.8)		61 (32.8)			125 (67.2)	

### Potential Benefits, Barriers, and Suggestions

From qualitative reports, there were 3 themes reflecting the potential benefits of digital peer support interventions: *shared experiences*, *social connection*, and *shared pain management solutions*. Table 5 provides a description of each theme identified and an example quote. Some participants suggested that they would benefit from the *shared experience* of CNCP and the ease of relating to others like them: “I think having people that understand the circumstances or what you go through each day would be beneﬁcial.”

Some participants reported that digital peer support could provide an opportunity for *social connection*, which may help with “Not feeling alone.” It could also provide an avenue for *shared practical pain management solutions*. This could involve problem-solving discussions as well as encouraging, motivating, and supporting each other to achieve goals: “Find things that have been helpful to others. From my experience of managing pain, I could perhaps help others who are struggling.”

Six themes reflected the potential barriers of peer support: negative focus on pain, judgement, lack of engagement, negative impact on mental health, privacy and security concerns, and unmet personal preferences. There were examples of how digital peer support may result in a negative focus on pain: “Everyone's pain is different, and I would become irritated by all the whinging.”

Participants were also concerned about *judgment* from others, and how it may create a competitive environment where people want to compare their experiences to others: “I would hate for it to be that people are trying to one up each other or believe that they are in more pain than someone else.”

Some reported personal and situational factors that could limit their engagement in digital peer support, including restricted access, limited time, and competing demands: “Being able to offer help when I feel I can't be reliable.”

Possible *negative impacts* on mental health were also reported including potential development or exacerbation of depression and anxiety symptoms: “I feel I would be taking other people's problems on board as well as my own and end up worrying about them as well as myself.”

*Concerns about privacy and security* of information in a digital forum were considered a barrier by some participants. *Personal preferences* for in-person connection could also be a barrier as some people prefer face-to-face rather than digital communication: “I haven't always found online groups very helpful as it feels isolating never having met the people and it also feels risky.”

Eight additional suggestions from participants were identified and are summarized in Textbox 1. These included: *moderation of the group*, *interest subgroups*, *professional-led activities*, *psychological strategies*, *links to professional pain resources*, *newsletter*, *motivational content*, *live streaming*, and *web-based meetups*.

**Table 5:** Description and examples of themes identified and frequency count.

Theme			Description		Example	Frequency, %^a^
**Benefits**						
	Shared experience	Ease of relating to others who share similar experiences to your own		“A shared understanding without judgement” “Having someone with similar experiences to share with”		38.5
	Social connection	Opportunity for social connection and feeling less alone		“Just connecting with others” “Not feeling alone”		27.8
	Shared pain management solutions	Opportunity to share ideas and solutions, problem solving with others, and motivating, or encouraging each other		“Sharing experiences and strategies for dealing with pain” “Talking to others who understand what it like to live with constant pain, and how to get some joy out of life”		27.0
**Barriers**						
	Negative focus on pain	People using the platform to discuss their pain in a negative way and not contributing constructively, and the negative impact this focus on pain can have		“Everyone's pain is different, and I would become irritated by all the whinging” “Often people use it for complaining and just want platitudes”		25.5
	Judgement	Experiencing negative comments from others about pain experience, feeling a lack of connection with pain community, or an environment that is competitive about severity of pain		“In the past I’ve found they can descend into a competition for “who is entitled to feel more pain” “I am not in as much pain as I was when ﬁrst diagnosed…sometimes it makes me feel ashamed or distant from the chronic pain community because I'm not ‘in pain’”		10.2
	Lack of engagement	Both personal and situation factors to engagement including, access, time, and motivational factors		“Interference with my work days” “Not feeling up to it physically”		20.4
	Negative impact on mental health	Experiencing a worsening of symptoms of depression or anxiety due to exposure to content on living with pain and concern over others met on the internet		“Taking onboard other peoples’ issues” “I was in a Facebook group, and I left it because it made me more anxious.” “Exposure to too much chronic pain not resolved”		17.2
	Privacy and security concerns	Concerns about privacy or lack of comfort with being on the internet		“Security of information. Privacy. Knowing who you’re talking to”		23
	Unmet personal preferences	Preference for in-person connection and lack of need for further support		“Online is very impersonal. Real connections and communications are made with people in person” “Not interested as have plenty of distractions and interests through radio, podcasts, audiobooks and sometimes TV”		12

^a^Benefits were summarized from 128 participants and barriers from 92 participants.

**Suggestions and examples for digital peer support (recommendations were summarized from 64 participants).**

**Moderation of the digital group**

- “Group would need to be adequately moderated by trained facilitators”

**Interest/subgroups groups for people with chronic noncancer pain**

- “Maybe groups that are for people with chronic pain. Like, this is a guitar group for people impacted by chronic pain.”

**Group activities led by professionals**

- “Clinical discussion on pain and management of the pain”

**Psychological strategies and activities**

- “I would like to have access to the management of chronic pain through mindfulness - specialised meditations…”

**Links to professional resources and relevant literature on pain management**

- “Further literature (current research) about chronic pain, pain management”

**Newsletter**

- “Email newsletter style with pain information and up to date medical information”

**Motivational or inspirational content on living with pain**

- “I would want to be around success stories, people that strive and survive and don’t let the chronic pain destroy them”

**Live stream content and/or online group meetups**

- “Meetups (online or RL) with access to discounted workshops for pain management (eg, Pilates classes for low back pain, tai chi and yoga classes as low impact exercise modalities)”

## Discussion

### Summary of Main Findings

The aim of this study was to examine interest in digital peer support interventions for CNCP, the role of pain self-efficacy and loneliness, and preferences for both content and delivery features. We also explored potential benefits, barriers, and suggestions for digital peer supported interventions. Our findings showed that there was interest in accessing digital peer-delivered interventions, with almost half of the sample indicating that they would access it if it was available. As expected, those who indicated interest in digital peer interventions reported both lower pain self-efficacy and greater loneliness than those who were not interested. Intervention content that incorporated education and links to health services and resources as well as delivery of support by peer coaches were the most frequently preferred intervention features. Three potential benefits were identified: *shared experience*, *social connection*, and *shared pain management solutions*. Five potential barriers were identified: *negative focus on pain*, *judgment*, *lack of engagement*, *negative impact on mental health*, *privacy and security concerns*, and *unmet personal preferences*. Finally, there were 8 suggestions from participants: *moderation of the group*, *interest subgroups*, *professional-led activities*, *psychological strategies*, *links to professional pain resources*, *newsletter*, *motivational content*, *live streaming*, and *web-based meetups*.

Interest in digital peer support was related to participants’ level of pain self-efficacy. Those who were less confident in managing day-to-day despite pain were more likely to be interested in accessing a digital peer support intervention. Low pain self-efficacy is a stronger predictor of pain-related disability than other psychological factors [4,9]. By contrast, higher pain self-efficacy is protective. It was found to be one of the only psychological factors assessed prior to the COVID-19 pandemic that predicted less disability post pandemic [33]. Participants reported that digital peer support interventions could promote pain self-efficacy. For example, *shared pain management solutions* included understanding how others manage daily tasks and sharing strategies for dealing with pain. These experiences may also provide opportunities for accessing informational support (eg, receiving advice). That is, participants viewed the intervention as a way to access new ideas and solutions for problems they were facing, in particular, from the perspective of those who are living with CNCP and who have personal experience of applying strategies in their own lives. Sharing lived experiences as part of digital peer-delivered interventions may be valuable not only for connecting with others but also for solution-focused strategies and empowered pain management, which could increase confidence in managing daily activities despite the pain. Interestingly, similar experiences were found in focus groups with participants in a face-to-face loneliness intervention (“Groups 4 Health”); the experience of collective problem-solving was nominated as one of the main things people valued about the program [34].

Those who reported higher levels of loneliness were more likely to be interested in accessing a digital peer-delivered intervention. This finding suggests that some individuals with CNCP have an unmet need for social connection. Participants highlighted that digital peer interventions could meet this need, providing a feeling of not being alone and a sense of “community.” Suggestions by participants for opportunities to connect with others who share common interests such as hobbies may support involvement in meaningful activities and contribute to a positive sense of self. It may also be that through this social connection they develop a “recovery identity” within the group that is collectively empowering [20].

Participants reported a high level of interest in receiving support from peer coaches. The role of peers in peer-delivered interventions can take many forms (eg, peer counselor, peer educator, peer support, peer case manager, and peer facilitator [35]). For people with CNCP, inclusion of peers in a peer coaching facilitator role may be particularly beneficial. Peer coaches are responsible for strengthening relationships between and among individuals that support them to set and achieve goals together [35]. It is possible that peer coaches provide a source of lived experience of *recovery*, offering patients hope for living a rich and meaningful life even with CNCP. Participants indicated a preference for inspirational or motivational content from peers, which suggests that peers can play not only a supportive role but act as role models for one another. More research is needed to determine if peer coaches enhance intervention engagement and effectiveness.

A preference for educational content and links to health services was found. Digital peer-delivered interventions could be well positioned to increase awareness of available resources and services as a “resource-hub.” Studies have shown that when peers adopt an educator role, intervention engagement can be improved [35]. Details on the preferred delivery of educational content included recommendations for live-streamed/digital group meet ups including professionally delivered group activities and talks delivered by experts. There was support for health professional guidance as part of peer-delivered interventions, including psychological strategies to improve coping skills. When therapist support is included as part of digital interventions, this has a positive impact on engagement and treatment effectiveness [36]. Future research could explore blended peer- and professional-led digital peer support interventions.

On average, approximately half of all participants indicated interest in each of the content and delivery features. However, there was overall less interest in a private chat function and completing questionnaires with feedback. Further investigation of specific preferences and how they may or may not be beneficial could guide how best to design and implement them. Providing options for digital (eg, mobile app) personalization may allow users to tailor aspects of the intervention to their specific needs and preferences. For example, participants may be able to opt out of the newsfeed function while still accessing the digital workshops or talks delivered by professionals. The degree of adaptability may increase the acceptability and usability of digital peer-supported interventions.

Our results suggest some participants may have been unsure of their interest in digital peer support. That is, a proportion of individuals who indicated a lack of interest in the broad idea of an intervention nevertheless indicated an interest in some of the proposed content and delivery features. It is possible that participants were initially unsure of their interest as relatively limited information about the digital peer support intervention was provided. For example, one participant commented, “Rather than yes/no – unsure – would depend on the group and group dynamics.” With further information about the intervention and context, it is possible that a greater number of individuals may be interested. Future research could explore any possible ambivalence. Supplementing web-based questionnaires with individual interviews and focus groups may also assist in exploring these issues. These findings may also indicate consensus on the needs of the CNCP community irrespective of personal interest in an intervention. This was evident in some of the responses provided by participants: “I wouldn’t be interested…however, I think they are good for people that need them” and “I don’t (know) whether I’d want to talk about chronic pain problems with others, I prefer to only talk about it with my GP. I think it is person(al) preference.” Seeking the perspectives of those who are not personally interested may elicit information relevant to the community at large and may also serve the function of supporting others.

A prominent barrier identified by participants was the potential negative impact of participating in a CNCP digital peer support intervention may have on mental health and well-being. This was a genuine concern. Studies have found a negative impact of exposure to the pain of others (vicarious pain) on pain outcomes [37]. Exposure to threatening information about pain is associated with reduced use of cognitive coping strategies [38]. It will be important to ensure patient safety in digital peer-delivered intervention groups. It is likely that professional- or peer-led involvement including moderation as suggested by participants could mitigate these concerns. To address the barrier of a possibly negative focus on pain, there was a suggestion for motivational or inspirational content on living with pain. The recommendation for inspirational or motivational stories from others living with pain may be beneficial for improving pain outcomes, as optimism and hope are associated with a positive adjustment to CNCP among other positive outcomes [39-41].

Recent reviews of patient-targeted mobile apps for pain self-management found that key stakeholders, including patients and health care professionals are not routinely engaged in the *design* of digital health interventions [42-44]. In this project, we advanced co-design of digital peer-delivered interventions to improve pain self-efficacy and social connectedness by examining both patient interest and their perspectives on intervention preferences as well as barriers and enablers to implementation in practice. Our study adds to the growing literature on co-design in digital health interventions [45] and extends research into the CNCP field.

We included a community sample of 186 patients. While there are no guidelines on the minimum number of key stakeholders or frequency/intensity of engagement that is needed in co-design, it is possible that our results may not generalize to the broader CNCP population or beyond the Australian context where patients have access to a relatively high standard of public health care. Continual and increased engagement of treatment-seeking patients in the subsequent steps of co-design and co-development of digital peer-delivered interventions could increase the customizability of the solution, to fit a wider range of users. We used yes or no questions to assess interest in a digital peer support intervention and its specific features. Future research may benefit from exploring nuanced perspectives by using continuous Likert scales to rate interest, or an additional response option of “Unsure.” Inclusion of additional qualitative methods (eg, interviews) may enrich the co-design process. In particular, further exploration of the goals that individuals would like to achieve may assist in tailoring intervention design to meet both their needs and enhance motivational factors. We examined 2 factors that were potentially associated with interest in digital peer-delivered interventions. There may be additional relevant factors, however, such as treatment expectations and related social connectedness concepts (eg, social group memberships [46]) that may influence patient interest. These require further consideration in future research.

Participants were recruited through advertisements on professional pain social media accounts and websites. The sampling method targeted people who had access to and were current users of the internet and social media. It is not clear whether interest in digital peer support interventions varies for those who may not be actively engaged in social media or currently have limited use of or access to the internet. Their perspectives could offer additional insights into the possible barriers and enablers to accessing digital social interventions. This study did not assess the participants’ level of digital literacy. Examining individuals’ skill or confidence in use of digital platforms may inform the design and impact of these digital interventions—for example, the level of support for technical questions or issues, or the relevance of tutorials or demonstrations. Future studies could also explore tailoring of peer support interventions for culturally and linguistically diverse populations. Social media sites can provide speech to text, translate text, as well as other accessibility modifications. Assessing the need for these features could be incorporated into the co-design process. Ensuring equitable access to digital social interventions is an important consideration in future work.

### Conclusion

Partnering with patients who have CNCP to identify their intervention preferences is critical to the co-design of impactful new and innovative digital treatments that better meet their needs and translate into everyday clinical care [25]. Patients were interested in digital peer-delivered interventions, and patients with lower pain self-efficacy and loneliness were more interested. It is possible that digital peer-delivered interventions tailored to these unmet needs may be beneficial in reducing the burden of living with CNCP. We identified preferences for intervention content and process of delivery as well as barriers and enablers, which could guide the future development of such programs. The next phases of co-design involves the generation of design concepts and prototyping [47]. Adopting a co-design approach has the potential to better address the needs of patients with CNCP, enhance translation of personalized digital peer-delivered interventions into practice and improve clinical effectiveness.

## Appendix

Multimedia Appendix 1 Purpose-built digital peer support intervention questions.

## Abbreviations

CNCP: chronic noncancer pain
REDCap: Research Electronic Data Capture
UCLS-8: UCLA Loneliness Scale

## Data Availability

Data access is restricted by our ethics protocol; however, the data sets generated during and analyzed during this study may be available from the corresponding author on reasonable request.
