# Supplementary material for: Interest in Digital Peer-Delivered Interventions and Preferences to Improve Pain Self-efficacy and Reduce Loneliness Among Patients With Chronic Pain: Mixed Methods Co-design Study
Source: JMIR Form Res. 2023 Apr 14;7:e41211. doi: 10.2196/41211 (PMC10148220; doi:10.2196/41211)
Supplement: Multimedia Appendix 1 [file formative_v7i1e41211_app1.docx]

**Supplementary Material
Purpose-built digital peer support intervention questions**

1. Is this peer support online group something you might like to access if it was available?
2. What would be some of the benefits of this online group?
3. What would be any downsides or challenges to this online group?
4. What features would you like to see to facilitate peer support? (please tick all that apply)
   1. Newsfeed
   2. Private chat option with a peer for 1-1 support
   3. Questionnaires about your pain and pain management experience for your own reflection with the possibility of sharing with others
   4. Monitoring and support by trained peer coaches who have experience with chronic pain
   5. Education about pain and pain management, including tips and self-care strategies
   6. Links to local health providers for extra support
5. An online peer support network is one idea. We would like to hear any other ideas you have about developing a peer support network for people with chronic pain. Please provide any comments or suggestions below.
